# Variation in target volume and centroid position due to breath holding during four‐dimensional computed tomography scanning: A phantom study

**DOI:** 10.1002/acm2.12692

**Published:** 2019-08-05

**Authors:** Yuta Miyamae, Mami Akimoto, Makoto Sasaki, Takahiro Fujimoto, Shinsuke Yano, Mitsuhiro Nakamura

**Affiliations:** ^1^ Division of Clinical Radiology Service Kyoto University Hospital Kyoto Japan; ^2^ Department of Radiological Technology, Radiological Diagnosis National Cancer Center Hospital Tokyo Japan; ^3^ Department of Radiation Oncology Kurashiki Central Hospital Okayama Japan; ^4^ Division of Medical Physics, Department of Information Technology and Medical Engineering, Human Health Sciences, Graduate School of Medicine Kyoto University Kyoto Japan

**Keywords:** 4DCT, breath‐holding time, scan mode, sorting algorithm

## Abstract

This study investigated the effects of respiratory motion, including unwanted breath holding, on the target volume and centroid position on four‐dimensional computed tomography (4DCT) imaging. Cine 4DCT images were reconstructed based on a time‐based sorting algorithm, and helical 4DCT images were reconstructed based on both the time‐based sorting algorithm and an amplitude‐based sorting algorithm. A spherical object 20 mm in diameter was moved according to several simulated respiratory motions, with a motion period of 4.0 s and maximum amplitude of 5 mm. The object was extracted automatically, and the target volume and centroid position in the craniocaudal direction were measured using a treatment planning system. When the respiratory motion included unwanted breath‐holding times shorter than the breathing cycle, the root mean square errors (RSME) between the reference and imaged target volumes were 18.8%, 14.0%, and 5.5% in time‐based images in cine mode, time‐based images in helical mode, and amplitude‐based images in helical mode, respectively. In helical mode, the RSME between the reference and imaged centroid position was reduced from 1.42 to 0.50 mm by changing the reconstruction method from time‐ to amplitude‐based sorting. When the respiratory motion included unwanted breath‐holding times equal to the breathing cycle, the RSME between the reference and imaged target volumes were 19.1%, 24.3%, and 15.6% in time‐based images in cine mode, time‐based images in helical mode, and amplitude‐based images in helical mode, respectively. In helical mode, the RSME between the reference and imaged centroid position was reduced from 1.61 to 0.83 mm by changing the reconstruction method from time‐ to amplitude‐based sorting. With respiratory motion including breath holding of shorter duration than the breathing cycle, the accuracies of the target volume and centroid position were improved by amplitude‐based sorting, particularly in helical 4DCT.

## 1 INTRODUCTION

Advances in technology have led to the development of high‐precision radiotherapy that can capture moving targets.^1^, ^2^, ^3^ To achieve this, the technology must recognize the motion of a target during the planning of radiation treatment. Four‐dimensional computed tomography (4DCT) has been used to obtain temporal and spatial information for a given target. Some studies have reported that 4DCT imaging can help determine the optimal irradiation field, including the planning target volume margin.^4^, ^5^, ^6^, ^7^ One such study showed that 4DCT is highly beneficial and should be used for radiation treatment planning when the tumor shows respiratory movement of more than 8 mm.^4^ In addition, Langner *et al*. reported that errors in target extraction and dose calculation may occur if motion artifacts in 4DCT images are not addressed.^8^

The two main 4DCT scanning modes are cine CT scanning mode (cine mode) and low‐pitch helical CT scanning mode (helical mode).^9^ In cine mode, image data are acquired with repeated couch movements. The images are multiple‐phase images, reconstructed at the same couch position into different phases based on the respiratory data. In this mode, respiratory data are used in the sorting process following image reconstruction. In helical mode, the image data are acquired while moving the couch with low helical pitch. The respiratory signal is added directly to the projection data during the image reconstruction process.

In clinical practice, several respiratory signals are used to reconstruct 4DCT images, such as tidal volume acquired with a spirometer^10^ and surface movement of the abdomen or chest wall acquired by real‐time position management (RPM),^11^ C‐RAD,^12^ and GateCT,^13^ or the pressure changes in a belt wrapped around the abdomen acquired with ANZAI^14^ and Bellows.^15^ These respiratory signals contain the amplitude and phase, and the 4DCT images are reconstructed using these data.

The amplitude‐based sorting algorithm recognizes end‐inspiration and end‐exhalation, and then determines their amplitudes according to the degree of respiration. Phase‐based sorting can be accomplished by time‐ and phase angle‐based sorting algorithms. The time‐based sorting algorithm uses uniformly spaced bins between two consecutive end‐inspiration phases. Meanwhile, the phase angle‐based sorting algorithm uses uniformly spaced bins between three respiratory phases (end‐inspiration, end‐exhalation, and the next end‐inspiration).

The 4DCT images are useful for defining moving targets. However, 4DCT cannot remove motion artifacts completely because image reconstruction algorithms assume that objects are stationary during scanning. Therefore, severe motion artifacts occur when 4DCT images are reconstructed from temporally inconsistent raw data. Some investigators have reported the effects of motion artifacts on the target volumes of 4DCT images in phantom studies.^16^, ^17^, ^18^ However, these studies did not account for the effects of irregular breathing patterns, such as unwanted breath holding. In clinical practice, patients sometimes unconsciously hold their breath during 4DCT scans, leading to artifacts due to missing raw data. Therefore, the moving target is inaccurately depicted in 4DCT images under breath‐holding conditions. In addition, for patients with irregular breathing patterns, including unwanted breath holding, alignment errors in the imaged target volume occur between planning CT and cone‐beam CT, which may result in under‐dosage to the target volume.^19^

This study investigated the effects of respiratory motion, including breath holding, on the target volume and centroid position of 4DCT images, according to different CT scanning modes and respiratory‐correlated sorting algorithms.

## 2 MATERIALS AND METHODS

### 2.1 Phantom and simulated respiratory motion

A QUASAR™ Programmable Respiratory Motion Phantom (Modus Medical Devices Inc., London, ON, Canada) was used to move a spherical object 20 mm in diameter along the longitudinal axis of the CT couch. The spherical object was moved by several simulated respiratory motions.

**Figure 1:**
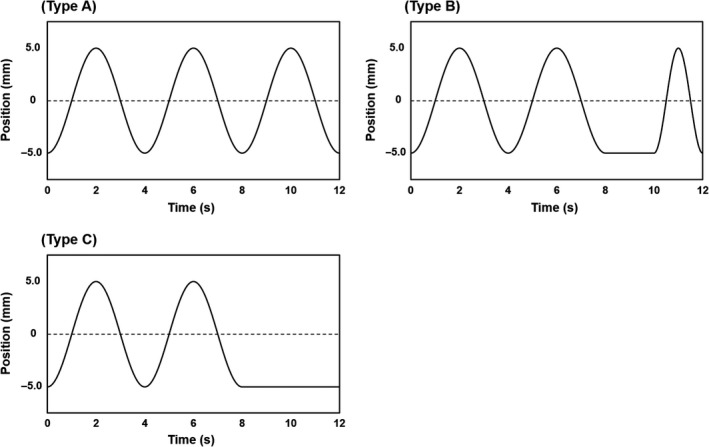
Simulated breathing patterns used to move the target object: Type A, squared trigonometric function; Type B, breathing pattern including a breath‐holding time of 2.0 s within a breathing cycle of 4.0 s (once every three cycles); and Type C, breathing pattern including a breath‐holding time of 4.0 s once every three cycles.

The respiratory motion was as follows:

(1) $$y\left(t\right)=2A\sin^{2}\left(\frac{\pi t}{T}-C\right)-A,$$

where *y*(*t*) is the target position at time *t*, *A* is the maximum amplitude of 5 mm, *T* is the motion period of 4.0 s, and *C* is the constant used to determine the starting phase of the respiratory motion. The following three respiratory motion patterns were used (as shown in Fig. 1):

- Type A, as described by formula (1).^20^
- Type B, as described by formulas (2) to (4) (*n *≥* *1):

(2) $$y\left(\text{t}\right)=2A\sin^{2}\left(\frac{\pi t}{T}-C\right)-A\;\left[3\left(n-1\right)T\leq \text{t}\leq \left(3n-1\right)T\right],$$

(3) $$y\left(t\right)=-A\;\left[\left(3n-1\right)T\leq \text{t}\leq 0.5\left(6n-1\right)T\right],$$

(4) $$y\left(t\right)=2A\sin^{2}\left(\frac{2\pi t}{T}-C\right)-A\;\left[0.5\left(6n-1\right)T\leq t\leq 3nT\right]$$

- Type C, as described by formulas (5) to (6) (*n *≥* *1):

(5) $$y\left(t\right)=2A\sin^{2}\left(\frac{\pi t}{T}-C\right)-A\;\left[3\left(n-1\right)T\leq t\leq \left(3n-1\right)T\right],$$

(6) $$y\left(t\right)=-A\;\left[\left(3n-1\right)T\leq t\leq 3nT\right].$$

In Types B and C, unwanted breath holding appeared once every three respiratory cycles.

### 2.2 4DCT data acquisition and image reconstruction process

The 4DCT images were acquired using two different CT scanners with different scanning modes: a cine mode CT scanner (LightSpeed RT16 11BW 46.3; General Electric Medical Systems, Waukesha, WI, USA) and a helical mode CT scanner (SOMATOM Definition AS VA48A and syngo CT VA48A; Siemens Medical Systems, Erlangen, Germany). The respiratory signal of moving targets was recorded using a RPM system (version 1.7, Varian) during CT scanning. To confirm reproducibility, measurements were repeated three times under each condition.

The 4DCT scan parameters in cine mode were as follows (Table 1): tube voltage, 120 kVp; tube current, 100 mA; gantry rotation time, 0.5 s/rot.; detector configuration, 16 × 1.25 mm collimation; interval between images, 0.25 s; scan duration (for each couch position), 6.0 s; and interscan delay, 2.0 s set to prevent marker vibration resulting from couch movement.^17^ The 4DCT images in cine mode were reconstructed with filtered back projection (FBP) using a standard kernel, with a slice thickness of 2.5 mm, increment of 2.5 mm, and field of view of 500 mm. Cine 4DCT images were reconstructed using the time‐based sorting algorithm. The obtained CT images and RPM data file were imported into an ADVANTAGE 4D workstation (AW 4.5; General Electric Medical Systems). ADVANTAGE 4D software was used to assign a phase to each CT slice according to the temporal correlation between the RPM data and the CT image, and 10 respiratory phase images acquired at regular intervals over a respiratory cycle were exported.

**Table 1:** 4DCT scan parameters for each CT scanner.

Parameters	Cine mode	Helical mode
Tube voltage (kVp)	120	120
Tube current (mA)	100	100
Gantry rotation time (s/rot.)	0.5	0.5
Detector configuration (mm)	16 × 1.25	64 × 0.6
Interval between images (s)	0.25	–
Scan duration (s)	6.0	–
Pitch factor	–	0.09

The 4DCT scan parameters in helical mode were as follows (Table 1): tube voltage, 120 kVp; tube current, 100 mA; gantry rotation time, 0.5 s/rot.; detector configuration, 64 × 0.6 mm collimation; and pitch factor, 0.09. The gantry rotation time and pitch factor were fixed in accordance with the vendor’s recommendations when the number of breaths per minute was 12 or more. Helical 4DCT images were reconstructed with FBP using a standard kernel (B31f); the slice thickness was 2.0 mm (increment of 2.0 mm), and the field of view was 500 mm. Helical 4DCT images were sorted using the time‐ and amplitude‐based algorithms. The obtained RPM data were imported into the CT scanner, and 4DCT images were reconstructed for 10 respiratory phase images using the sorting algorithms. We did not employ the phase angle‐based sorting algorithm because it uses raw data and does not take into account the breath‐holding time during image reconstruction, as with the time‐based sorting algorithm.

### 2.3 Target volume and centroid position analyses

The 4DCT images were imported into a commercial three‐dimensional radiation treatment planning system (Eclipse 13.7; Varian, Palo Alto, CA, USA). The spherical object was detected by semiautomatic extraction according to the CT threshold, which was taken as the CT value when the volume of the spherical object matched the reference value (V_0_: 4.19 ml) in the stationary image. Note that V_0_ was calculated as V_0_ = (4/3) × *π* × *r* ^3^, where *r* is the radius of the spherical object. The CT thresholds used for semiautomatic extraction of spherical objects were 166 and 174 HU in cine mode and helical mode, respectively. The object was automatically extracted and the target volume and centroid position in the craniocaudal direction were measured.

Shifting of the time series was required, as shown by comparison of the measured centroid positions in cine mode with those in helical mode; this was because the image reconstruction algorithms employ different time scales depending on the vendor. For each phase reconstructed using helical mode 4DCT, the center phases of the images differed from those reconstructed using cine mode 4DCT. As one example of 50% phase, the temporal center of the 50% phase is at the 50% +0.125 s position; therefore, the reference and measured helical mode centroid positions were converted from phase to time series data according to a reference value of respiratory motion of 0.125 s for ease of comparison between the time‐ and amplitude‐based sorting algorithms.

## 3 RESULTS

### 3.1 Target volume analyses

**Figure 2:**
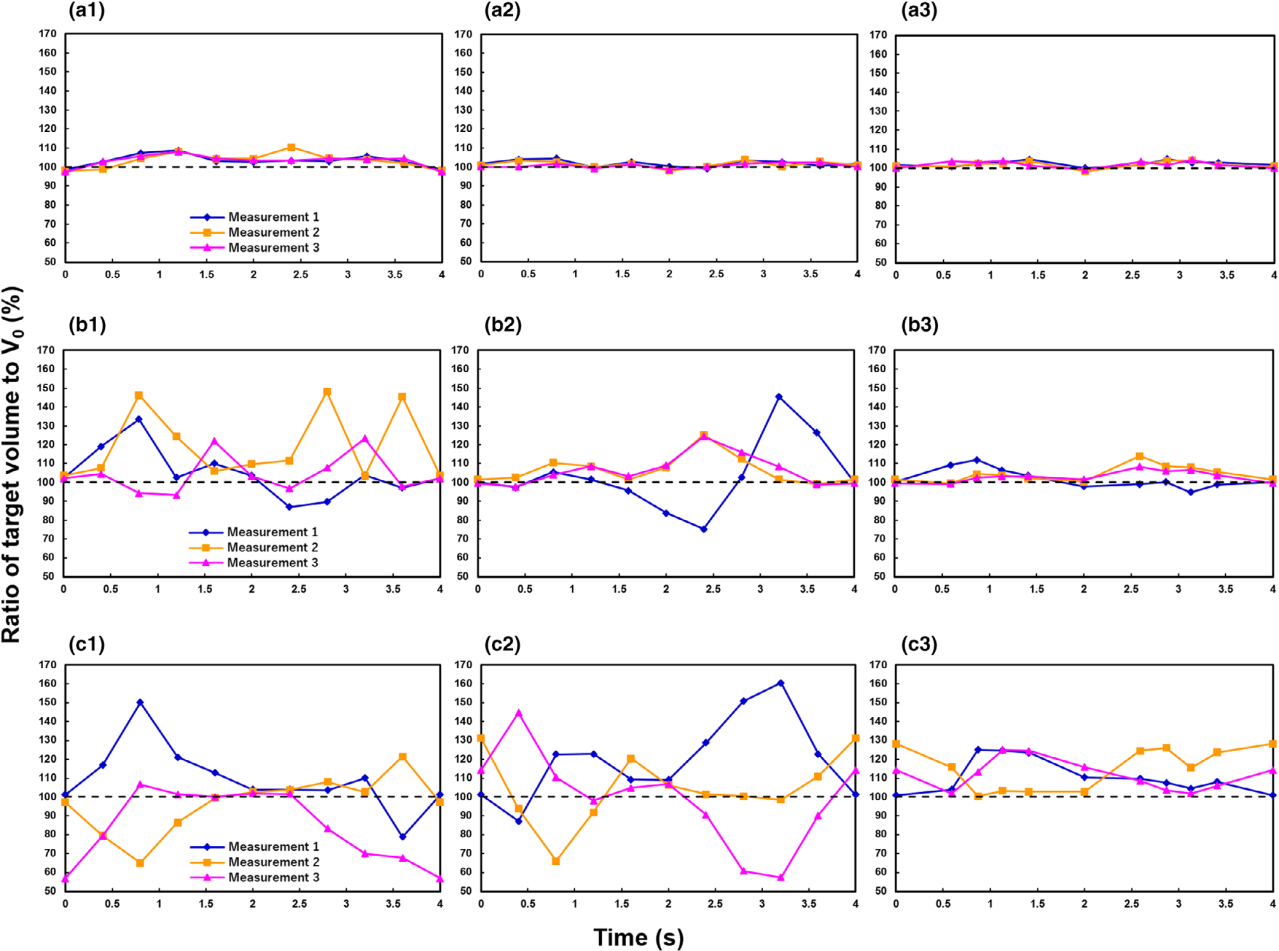
Ratio of target volume to the reference value (V_0_): (a) Type A respiratory motion, (b) Type B respiratory motion, and (c) Type C respiratory motion. (1) Cine four‐dimensional computed tomography (4DCT) with time‐based sorting, (2) helical 4DCT with time‐based sorting, and (3) helical 4DCT with amplitude‐based sorting.

The ratios of target volume to V_0_ of the three reconstruction methods are shown in Fig. 2 for the various respiratory motion patterns. With Type A respiratory motion, the root mean square errors (RMSEs) between the reference and imaged target volume were 4.87%, 2.12%, and 2.66% in the time‐based images in cine model, time‐based sorting images in helical mode, and amplitude‐based images in helical mode, respectively. With Type B respiratory motion, the values were 18.8%, 14.0%, and 5.53%. For helical mode images, variation in target volume was reduced by changing the reconstruction method from time‐ to amplitude‐based sorting. Type C respiratory motion was associated with the greatest variation in target volume for all reconstructions based on other respiratory motion types. The RMSEs in target volume with Type C respiratory motion were 19.1%, 24.3%, and 15.6% in time‐based images in cine mode, time‐based images in helical mode, and amplitude‐based images in helical mode, respectively.

### 3.2 Target centroid position analyses

**Figure 3:**
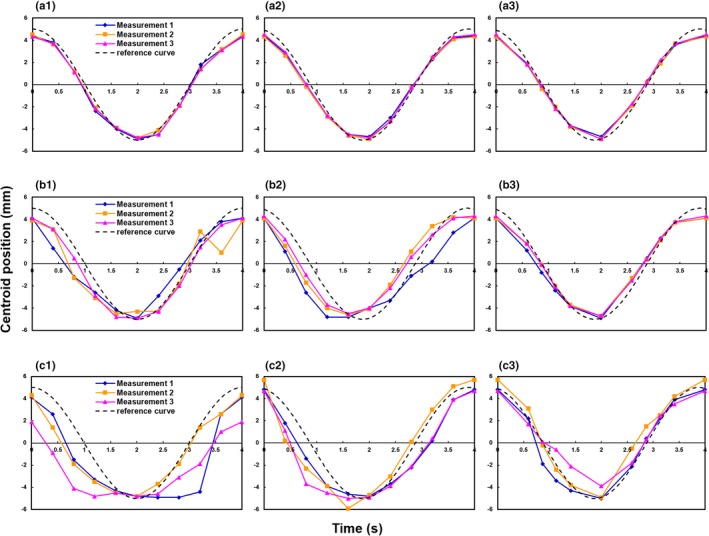
Centroid position in the craniocaudal direction: (a) Type A respiratory motion, (b) Type B respiratory motion, and (c) Type C respiratory motion. (1) Cine 4DCT with time‐based sorting, (2) helical 4DCT with time‐based sorting, and (3) helical 4DCT with amplitude‐based sorting.

**Figure 4:**
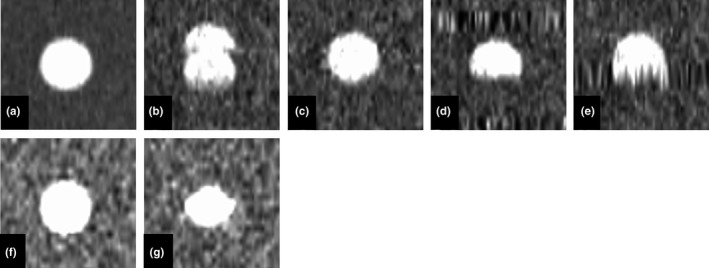
Example coronal images of the spherical object and helical 4DCT images of the spherical object: (a) spherical image scanned without movement, (b) helical 4DCT with time‐based images for Type B respiratory motion (80% phase), (c) helical 4DCT amplitude‐based images for Type B respiratory motion (80% phase), (d) helical 4DCT time‐based images for Type C respiratory motion (80% phase), (e) helical 4DCT amplitude‐based images for Type C respiratory motion (80% phase), (f) cine 4DCT time‐based images for Type B respiratory motion (80% phase), and (g) cine 4DCT time‐based images for Type C respiratory motion (80% phase).

Figure 3 shows the centroid positions of the three reconstruction methods for each respiratory motion pattern. With Type A respiratory motion, the RMSEs between the reference and imaged centroid position were 0.48, 0.42, and 0.29 mm in time‐based images in cine mode, time‐based images in helical mode, and amplitude‐based images in helical mode, respectively. With Type B respiratory motion, the values were 1.29, 1.42, and 0.50 mm. With Type C respiratory motion, they were 2.52, 1.61, and 0.83 mm. The amplitude‐based images in helical mode showed the highest positional accuracy (Fig. 3). Particularly, with Type B respiratory motion, the reproducibility of the centroid position was improved by amplitude‐based reconstruction. Figure 4 shows examples of spherical object images captured in helical mode with Type B and C respiratory motion.

## 4 DISCUSSION

We used two different types of CT scanner to investigate the impact of CT scanning modes and respiratory‐correlated sorting algorithms on target volume and centroid position accuracy in 4DCT images acquired using a moving phantom. To the best of our knowledge, this is the first study to assess these metrics in 4DCT images in the context of respiratory motion patterns, including breath holding. The results suggest that if the breath‐holding time does not exceed the average respiratory cycle time, the target volume and centroid position can be reproduced with high accuracy in amplitude‐based reconstructions. Meanwhile, target volume and centroid position accuracy could not be maintained, regardless of the scan mode or reconstruction method, when breath holding occurred throughout the 4DCT scan.

With regular respiratory motion without breath holding (Type A), the centroid position of amplitude‐based images in helical mode was more accurate than time‐based images in cine mode and helical mode. The amount of projection data used for 4DCT image reconstruction varies among CT scanners. Respiratory data are added to the reconstructed images in cine mode. On the other hand, in helical mode, only the raw data are extracted, and then the 4DCT images are reconstructed. This results in better temporal resolution because, in helical mode, 4DCT images do not contain unnecessary temporal phase data. In general, the effective temporal resolution of a scan using a short scan reconstruction is equal to half the gantry rotation time in helical mode.^21^ We did not discuss the temporal interval for the Siemens scanner because the operators cannot change the temporal interval. The RMSE between the reference and target volume with Type A respiratory motion were smaller in time‐based images in helical mode than in time‐based images in cine mode. We consider that helical mode has superior in temporal resolution to cine mode, which would cause less motion artifacts. In addition, whereas the time‐based sorting algorithm recognizes only inspiration phases, and then divides the phase accordingly, the amplitude‐based sorting algorithm recognizes both inspiration and exhalation phases. Therefore, amplitude‐based sorting yields a more accurate centroid position.

Previous studies have reported various artifacts in 4DCT images, including blurring, as well as duplicated, overlapping, and incomplete structures.^16^, ^22^, ^23^ Among these, overlapping and incomplete structure artifacts (Fig. 4) occurred more often with the time‐based sorting algorithm in this study, and as expected, the degrees of error in target volume and centroid position were increased compared to the amplitude‐based sorting algorithm. The major difference between the time‐ and amplitude‐based sorting algorithms lies in the use of raw data, including breath‐holding time, for 4DCT image reconstruction: the amplitude‐based sorting algorithm does not use all acquired raw data for the duration of constant amplitude the respiratory, and therefore expresses respiratory motion more accurately than the time‐based algorithm.

With respiratory motion including breath holding of shorter duration than the breathing cycle (Type B), the target volume and centroid position accuracies were improved by amplitude‐based reconstruction. When one inspiration and one exhalation are present in the average respiratory cycle, amplitude‐based reconstruction can be successful. However, it should be noted that the target volume was overestimated when there was a motion artifact and respiration occurred earlier than normal within the respiratory cycle. If a respiratory pattern, such as Type C, occurs during 4DCT scanning, accuracy is not likely to be improved by changing scan mode or reconstruction method. In particular, zonal truncation artifacts, which are specific to helical 4DCT, occurred with Type C respiratory motion due to undersampling of the respiratory cycle (Fig. 4). The target volume was affected by the extent of the zonal truncation artifacts and tended to be overestimated. In addition, the imaged centroid position was different from the reference position due to the artifacts. When respiration patterns, such as Type C, are encountered during 4DCT scanning, rescanning should be considered, although this could increase the radiation dose delivered to the patient. Therefore, it is important to encourage radiological technologists engaged in 4DCT scanning to acquire reproducible and regular respiration data during CT data acquisition.

Our study had several limitations. First, the maximum amplitude of the simulated respiratory motion was low, at only 5 mm. However, if the maximum amplitude of the respiratory motion is 10 mm or more, the accuracy of the target volume and centroid position decreases because motion artifacts become significant.^17^ Second, simplified respiratory patters were used in this study, while actual respiratory patterns are more complicated (Fig. 1). In addition, such motions would involve centroid movement coupled with couch motion in helical scanning mode due to interplay between couch speed and phantom motion. Therefore, it should be noted that larger errors would occur in clinical practice than observed in our study. Third, the two CT scanners used could not reconstruct images of the same slice thickness (2.5 mm in cine mode and 2.0 mm in helical mode, appropriate for clinical radiotherapy planning). The impact of the partial volume effect may differ by slice thickness. However, the size of the spherical object used in this study was large enough compared to the difference in spatial resolution, so we believe that the difference would not have affected our results.

## 5 CONCLUSION

We investigated the effects of respiratory motion, including breath holding, on target volume and centroid position in 4DCT images acquired with different CT scanning modes and respiratory‐correlated sorting algorithms.

Our results suggest that 4DCT images acquired in helical mode depict the target volume and centroid position more accurately than cine 4DCT. In addition, with respiratory motion including breath holding of shorter duration than the breathing cycle, the accuracy of the target volume and centroid position are improved by amplitude‐based sorting, particularly in helical 4DCT.

## CONFLICT OF INTEREST

No conflict of interest.
